# Analysis of human hippocampal volumetry in relation to pattern separation ability in healthy young subjects

**DOI:** 10.1002/brb3.1878

**Published:** 2020-10-23

**Authors:** Ryoichi Usugi, Masahiko Nishimura, Shogo Ishiuchi

**Affiliations:** ^1^ Department of Neurosurgery Graduate School of Medicine University of the Ryukyus Okinawa Japan

**Keywords:** dentate gyrus, hippocampal subfields, hippocampal‐amygdaloid transition area HATA, pattern separation, volumetric MRI

## Abstract

**Introduction:**

Hippocampal dentate gyrus related to pattern separation has attracted attention as an area for neurogenesis. However, the associations between the pattern separation and the volumes of hippocampal subfields in humans remain unknown.

**Methods:**

58 young adults were examined the memory task (pattern separation, pattern completion) and the hippocampal volumes. Subjects were asked to determine whether the visual image is a new stimulus, or a similar but different stimulus (pattern separation), or the same stimulus (pattern completion), compared to preceding stimuli, and response time and correct response were measured. The volumes of the whole brain, hippocampus 6 subfields and perihippocampus 5 subfields, were measured using FreeSurfer 6.0.

**Results:**

Negative associations between the pattern separation task and the volumes of whole brain areas were found in bilateral cerebellar cortex, fourth ventricle, left hippocampus, left thalamus, left ventral diencephalon, and brainstem. Simple linear regression analysis revealed a significant association with the left hippocampal‐amygdaloid transition area only, while no significant associations were found in any of the subfield volumes when adjusted with covariates.

**Conclusions:**

The principle “bigger is better”—an idea that the larger the volume the better the function—could not be applied to the relation between the pattern separation ability and the dentate gyrus.

## Graphical abstract

The pattern separation ability which distinguishes similar but different stimuli is the kernel of our memory. The novelty and the social significance lies in its evaluation of the associations between the pattern separation ability related to neurogenesis and the volumes of the subfields including the dentate gyrus of the hippocampus in healthy young adults. There were no significant causal associations when adjusted with covariates.

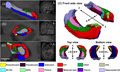

## 1 INTRODUCTION

Neurogenesis is reported to be continued over a lifetime even in the adult brain (Boldrini et al., 2018; Eriksson et al., 1998; Moreno‐Jiménez et al., 2019) and take place in the subventricular zone and the subgranular zone of the hippocampal dentate gyrus (DG). Neurogenesis in DG has been reported to be associated with the pattern separation, an ability to detect similar but different characteristics, in rodents (França et al., 2017; Leutgeb et al., 2007) and fMRI analyses on humans found that the blood oxygenation level‐dependent effect (BOLD) in the hippocampal DG was correlated with the activity of Cornu ammonis (CA) 4, anterior mid‐cingulate gyrus, and lateral cerebellum (Bakker et al., 2008; Shiroma et al., 2015). On the other hand, the function of reproducing neural patterns with complete information from incomplete information and recalling the memory after memory learning is termed the pattern completion, whose function is reported to be located in the hippocampal CA3 region for rodents (Rolls, 2013), and associated with the CA1 region in humans (Bakker et al., 2008). Further, a study that used genetically engineered animals demonstrated that younger granular cells in the DG are involved in the pattern separation, while older cells are associated with the pattern completion, suggesting that as young neurons get old, they gradually switch their roles from the pattern separation to pattern completion (Nakashiba et al., 2012). It has also been reported that adult rats in an enriched environment showed an increase in the number and volume of newborn cells in the subgranular layer of the DG, as well as the elongation of dendrites and proliferation of glial cells (Kempermann et al., 1997), demonstrating that there is plasticity in the hippocampal volume.

MRI is a useful tool for the research in the neural plasticity of human brain that can noninvasively quantify the structure of the whole brain (May, 2011). There are quite a few interesting reports from the point of view of neural plasticity regarding the associations between the brain structure and memory in humans (Draganski et al., 2006; Golestani et al., 2002; May, 2011). It has been reported that taxi drivers in London possess excellent spatial memory, and the greater the spatial memory, the greater the volume of the hippocampal tail, suggesting that experience may cause structural and plastic changes in the brain (Maguire et al., 2000). In a study that uses diffusion tensor imaging (DTI) which can quantitatively assess the white matter tract integrity to analyze healthy subjects, microstructural changes were observed in the language network associated with reading comprehension along with the improvement on the task performance after just 2 hr of vocabulary learning (Hofstetter et al., 2017). It has been reported that even a 45‐min spatial route learning task was associated with the improved behavioral performance and microstructural changes in the posterior‐dorsal DG of the left hippocampus (Keller & Just, 2016). A systematic review of 33 articles that analyzed the relationships between the hippocampal volume and memory performance in healthy human subjects (Van Petten, 2004) reported that, with regard to the association between the hippocampal volume and memory performance estimated from MRI, the “bigger is better” hypothesis cannot be supported for elderly people, and for young subjects, surprisingly, the results rather supported the “smaller is better” hypothesis, indicating that healthy subjects showed high variability. Doxey et al the manual segmentation method based on MRI data to calculate the volumes of hippocampal subfields in order to investigate the associations between the pattern separation ability and the volumes of hippocampal subfields, and reported that CA3 and DG in the left hippocampus were positively associated with the pattern separation performance in both young and elderly subjects (Doxey & Kirwan, 2015). There are two MRI‐based hippocampal volumetric analyses: the manual segmentation method and the automation segmentation method. The issue with the former, it has been suggested, is that it takes a huge amount of time, and the data are arbitrary (Van Leemput et al., 2009). In this research, we used in FreeSurfer 6.0. The advantage of using FreeSurfer 6.0 was the first time enabled automated segmentation into the hippocampus of the 6 subfields as CA1, CA2/3, CA4, granule cell layer of dentate gyrus (GC‐DG), molecular layer (ML) and subiculum, and the perihippocampus of the 6 subfields as tail, presubiculum, parasubiculum, hippocampal fissure, fimbria and hippocampal‐amygdaloid transition area (HATA). Worker et al. tried to validate the test–retest reliability of FreeSurfer 6.0 with standard resolution T1 in a study of healthy elderly subjects and patients with Alzheimer's disease and demonstrated that it was highly reliable with the intraclass correlation coefficient (ICC) of 0.9 or greater in all subfields except for hippocampal fissure and fimbria, and concluded that FreeSurfer 6.0 is a robust and useful method (Worker et al., 2018). In view of these considerations, in this study, we tried to estimate the subfield volumes of hippocampus and perihippocampus using the FreeSurfer 6.0 auto segmentation method in young subjects and clarify whether there are any functional or structural associations between the pattern separation, which reportedly bore functional associations with hippocampal subfields, and DG, and between the pattern completion and CA3 & CA1.

## 2 MATERIALS AND METHODS

### 2.1 Subjects

The subjects enrolled in this study were 58 healthy volunteers (mean age 25.0 ± 4.4 years, range 18 to 40 years; 31 males 24.5 ± 3.5 and 27 females 25.5y ± 5.2). All the subjects were right‐handed according to the Edinburgh Handedness Inventory (Oldfield, 1971). None of the subjects had any signs or history of neurological or psychological diseases. All subjects provided informed consent for this investigation. The study has approved by the ethical committee of the University of the Ryukyus.

### 2.2 Behavioral memory task

Details of the method of the fMRI task were described in a previous study (Shiroma et al., 2015). In brief, the memory test used fully randomized tasks with 108 trials based on an explicit three‐alternative forced‐choice task, including 44 unrelated novel (“New”), 16 repeated (“Same,” for pattern completion), and 16 similar (“Lure,” for pattern separation) stimuli consisting of color photographs of common objects. Using Presentation^®^ software (Neurobehavioral Systems, Inc., Austin, Texas, USA), each visual stimulus was presented to participants through the goggles display under computer control (Resonance Technologies, Inc., Salem, Massachusetts, USA) for 2,500 ms, with a 0–1,000 ms interstimulus interval to prevent adaptive stimulus response. Subjects were required to press a button: red for a new object, blue for a repeated object, or green for a lure object. Responses and reaction times were recorded in a button box (Current Designs, Inc., Philadelphia, Pennsylvania). We calculated the correct response rate (CRR) for lure and same task responses and defined them as a behavioral memory task score.

### 2.3 Structural MRI acquisition

MRI images were acquired using a 3‐T MRI scanner (Discovery MR 750; GE Healthcare, Waukesha, Wisconsin, USA) with a 32‐channel head coil. The structural three‐dimensional (3D) T1‐weighted MRI images were acquired using a spoiled gradient‐recalled echo (SPGR) sequence to obtain standard resolution 1‐mm slice thickness scans with the following parameters: repetition time 6.9 ms, echo time 3 ms, flip angle 15°, matrix size 256 × 256, and field of view 256 × 256 mm. A high‐resolution T2 fast spin echo (T2 FSE) sequence (repetition time 4,300 ms, echo time 92 ms, matrix size 512 × 512, field of view 192 × 192, in‐plane resolution 0.375 × 0.375 mm^2^, 23 slices, 3‐mm thickness, 0‐mm space) was obtained. T2 FSE structural images were acquired in an oblique coronal plane perpendicular to the long axis of the hippocampus. Almost the entire hippocampus (head, body, and tail) was included in the 23 slices.

### 2.4 Volumetric analysis

All T1‐weighted images data were processed using the freely available software FreeSurfer version 6.0 (http://surfer.nmr.mgh.harvard.edu). We analyzed the data using the fully automatic reconstruction (“recon‐all”) function in FreeSurfer 6.0 for volumetric segmentation, including motion correction, Talairach transform computation, intensity normalization, skull stripping, cortical area volumetric labeling, white matter segmentation, gray/white matter tessellation, and surface extraction. The processing included removal of nonbrain tissue and segmentation of the cortical gray matter, white matter, and subcortical volumetric structures. Neuroanatomical areas were estimated by automatically assigning labels to each voxel in the MRI volume based on probabilistic information from the manually labeled training set (Fischl et al., 2002). Before analysis of the hippocampal subfield, we extracted data of each area's volume, segmented by automated segmentation for subcortical structures (Aseg). We then applied simple linear regression analysis for memory task scores in the areas’ segmented volumes. Vessels and the fifth ventricle were excluded from the analysis due to estimation error.

**Figure 1:**
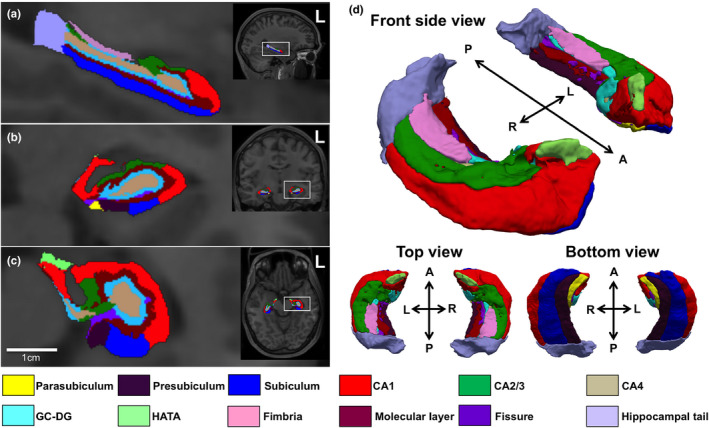
The subfields of hippocampus and perihippocampus in healthy subject. Note, the subfields of hippocampus and perihippocampus are shown in sagittal (a), coronal (b), axial (c), and (d) 3D views, respectively, for a healthy subject. The subfields are indicated each color. *Yellow, deep purple, blue, red, green, gray, light blue, light green, pink, brown, purple,* and *light purple* indicates the parasubiculum, presubiculum, subiculum, cornu ammonis (CA) 1, CA 2/3, CA4, granule cell layer of dentate gyrus (GC‐DG), hippocampal–amygdaloid transition area (HATA), fimbria, molecular layer, fissure, and hippocampal tail, respectively. A, anterior; P, posterior; L, left; R, right

Then, we applied automated analyses of the subfield of the hippocampus using the probabilistic atlas and a modified version of Van Leemput's algorithm (Van Leemput et al., 2009) to segment the hippocampus and perihippocampus (Iglesias et al., 2015). We extracted the volumes of the whole hippocampus including hippocampus and perihippocampus, as well as of the hippocampus 6 subfields and the perihippocampus 5 subfields for each hemisphere, excluding the hippocampal fissure. We defined the 6 subfields of hippocampus as CA1, CA2/3, CA4, GC‐DG, ML, and subiculum, and the 5 subfields of perihippocampus area as tail, presubiculum, parasubiculum, fimbria, and HATA. The hippocampal fissure has been reported as being poorly reproducible, in contrast to the reliability of the 6 subfields of hippocampus and the 5 subfields of perihippocampus segmented using FreeSurfer 6.0 with standard resolution T1 data may serve as reliable (Whelan et al., 2016). For the above reasons, we analyzed the 6 subfields of hippocampus and the 5 subfields of perihippocampus excluding the hippocampal fissure to evaluate only brain structures (Figure 1). In the subfields analysis included the 6 subfields of hippocampus and the 5 subfields of perihippocampus, standard resolution T1 was analyzed in all subjects, high‐resolution T2 data were additionally analyzed in 47 all of 58 subjects. The volume data calculated from the standard resolution T1 and the high‐resolution T2 showed a high correlation with significance level less than 0.5% in all the subfields analyzed by correlation analysis and confirmed the reliability of both (Data S1). All postprocessing data were visually checked for segmentation accuracy by trained operators. The visual quality check focused on both overall image quality and accuracy of the whole brain volumetric segmentations, such as skull strip errors, segmentation errors, intensity normalization errors, pial surface misplacement, and topological defects. No manual interventions on the data were performed.

Total intracranial volume (TIV) estimated by FreeSurfer 6.0 was adopted as a covariate for brain volume correction.

### 2.5 Statistical analysis

Continuous variables except age and same score, showed standard distribution at *p*> .05 with the Kolmogorov–Smirnov test, we used the Welch *t* test and the Mann–Whitney U test for the variable of gender difference.

We applied an analysis of covariance (ANCOVA) to find differences for the gender groups adding TIV and age as covariates, in the volumes for the whole hippocampus and the each subfield including hippocampus 6 subfields and perihippocampus 5 subfields. To examine whether the behavioral memory task score (lure and same correct response rate percentages) was associated with the segmented whole brain, whole hippocampus, and subfield volumes of hippocampus and perihippocampus in healthy subjects, we first used simple linear regression analysis between the behavioral task scores and brain volumes of whole brain, whole hippocampus and subfield volumes with the 6 subfields of hippocampus and the 5 subfields of perihippocampus, for bilateral, left, and right. Then, multiple regression analyzes were performed with the whole hippocampus volumes and subfield volumes separately, TIV, age, and gender as explanatory variables and the behavioral variable as the dependent variable—for example, introducing the lure score with dependent variables, left CA1 volume, TIV, age, and gender as explanatory variables into a model on the R statistical command line: lm (lure score ~ left CA1 volume + TIV + age + gender).

The Benjamini–Krieger–Yekutieli method (Benjamini & Yekutieli, 2001) was used for multiple comparison correction for simple linear regression analysis and multiple regression analysis, across 12 comparisons in hippocampus analysis (whole hippocampus and 11 subfield region) and 46 comparisons in whole brain analysis.

All statistical analyses were conducted with an alpha level of *p* = .05, using EZR (Saitama Medical Center, Jichi Medical University), which is a graphical user interface for R (Version 3.0.2, www.R‐project.org). More precisely, EZR is a modified version (“easy R”) of the R commander designed to add statistical functions frequently used in biostatistics (Kanda, 2013).

## 3 RESULTS

### 3.1 Gender differences in the correct response rate (CRR) and all subfield volumes

The mean CRR for younger people aged 40 or lower was 50.1 ± 18.2% in the lure task, and it was 89.1 ± 10.4% in the same task. There were no gender differences in the CRR in both the lure task and same task, or response time to each stimulus, lure, and same (Table 1). A comparison between males and females in the subfield volumes of hippocampus and perihippocampus revealed that the left GC‐DG (*p* = .01, *F* value = 7.1) and the left fimbria (*p* = .02, *F* value = 5.5) showed gender difference, no significant differences were noted in other regions (Table 2). Analysis using additional high‐resolution T2 data also showed a significant gender differences with left GC‐DG (*p* = .05, *F* value = 4.0) and left fimbria (*p* = .02, *F* = 6.0) (Data S2).

**Table 1:** Behavioral results of the lure and same task in all study subjects (*n* = 58)

	Male	Female	Statistic value		p value	
	Mean	Mean				
*n*	31	27	0.28	†	0.60	*n*.s
Age (years)	24.5 (3.5)	25.5 (5.2)	373.0	‡	0.48	*n*.s
Lure score (%)	49.9 (21.3)	50.3 (14.2)	−0.07		0.95	*n*.s
Same score (%)	89.7 (10.2)	88.3 (10.7)	447.5	‡	0.65	*n*.s
Lure response time (sec)	1.40 (0.17)	1.4 (0.25)	0.15		0.89	*n*.s
Same response time (sec)	1.21 (0.17)	1.2 (0.23)	0.06		0.95	*n*.s

Note

Data expressed as mean ± standard deviation (*SD*), the number of subjects (*n*), and not significant (*n*.s). Chi‐square test†, Mann–whitney U test‡, Welch *t* test.

**Table 2:** Characteristic of the subfield volumes of hippocampus and perihippocampus segmented using standard resolution T1

Left volume	Male		Female	
	(*n* = 31, 24.5y ± 3.5)		(*n* = 27, 25.5y ± 5.2)	
	mean mm^3^ (95% CI; *SD*)	Vol/ TIV 10^4^ (95% CI; *SD*)	mean mm^3^ (95% CI; *SD*)	Vol/ TIV 10^4^ (95% CI; *SD*)
Whole hippocampus	3,624.9 (3,526.3, 3,723.5; 268.8)	21.44 (20.77, 22.11; 1.83)	3,387.6 (3,291.7, 3,483.5; 242.4)	22.45 (21.85, 23.05; 1.51)
Subiculum	456.8 (438.7, 474.8; 49.3)	2.70 (2.59, 2.82; 0.32)	433.6 (419.7, 447.5; 35.1)	2.88 (2.77, 2.98; 0.26)
Presubiculum	328.1 (315.9, 340.2; 33.1)	1.94 (1.86, 2.02; 0.22)	315.5 (305.1, 325.9; 26.2)	2.09 (2.01, 2.17; 0.20)
Parasubiculum	57.9 (54.0, 61.8; 10.7)	0.34 (0.32, 0.37; 0.07)	56.2 (51.9, 60.5; 10.8)	0.37 (0.34, 0.40; 0.08)
Hippocampal tail	583.7 (550.9, 616.4; 89.2)	3.45 (3.24, 3.66; 0.57)	578.0 (547.7, 608.2; 76.5)	3.83 (3.64, 4.02; 0.48)
Molecular layer	597.0 (580.2, 613.9; 45.9)	3.53 (3.42, 3.64; 0.31)	557.4 (539.8, 575.0; 44.4)	3.69 (3.59, 3.80; 0.27)
GC‐DG *	313.1 (304.2, 322.0; 24.3)	1.85 (1.79, 1.92; 0.17)	281.9 (273.4, 290.5; 21.7)	1.87 (1.81, 1.93; 0.14)
CA1	662.9 (642.1, 683.8; 56.8)	3.92 (3.79, 4.05; 0.37)	612.3 (587.3, 637.3; 63.2)	4.05 (3.91, 4.19; 0.35)
CA2/3	209.3 (201.0, 217.6; 22.6)	1.24 (1.19, 1.29; 0.13)	186.9 (178.9, 194.9; 20.2)	1.24 (1.19, 1.28; 0.11)
CA4	264.2 (255.9, 272.5; 22.6)	1.56 (1.51, 1.62; 0.15)	239.9 (232.2, 247.5; 19.3)	1.59 (1.54, 1.64; 0.12)
fimbria *	89.7 (83.2, 96.2; 17.8)	0.53 (0.49, 0.57; 0.10)	72.0 (65.3, 78.6; 16.8)	0.48 (0.43, 0.53; 0.12)
HATA	62.4 (59.4, 65.3; 8.0)	0.37 (0.35, 0.38; 0.04)	54.0 (51.1, 56.9; 7.3)	0.36 (0.34, 0.38; 0.05)
Right volume	Male		Female	
	mean mm^3^ (95% CI; *SD*)	Vol/ TIV 10^4^ (95% CI; *SD*)	mean mm^3^ (95% CI; *SD*)	Vol/ TIV 10^4^ (95% CI; *SD*)
Whole hippocampus	3,781.9 (3,651.3, 3,912.5; 356.1)	22.35 (21.58, 23.11; 2.09)	3,510.0 (3,400.8, 3,619.2; 276.0)	23.29 (22.46, 24.12; 2.10)
Subiculum	474.5 (454.3, 494.7; 55.1)	2.81 (2.68, 2.94; 0.35)	435.9 (419.0, 452.8; 42.8)	2.89 (2.76, 3.03; 0.34)
Presubiculum	324.6 (309.6, 339.7; 41.1)	1.92 (1.83, 2.00; 0.23)	302.5 (290.4, 314.6; 30.5)	2.01 (1.91, 2.11; 0.26)
Parasubiculum	58.7 (54.5, 63.0; 11.5)	0.35 (0.32, 0.37; 0.06)	52.1 (48.7, 55.4; 8.5)	0.35 (0.32, 0.37; 0.06)
Hippocampal tail	616.0 (587.9, 644.2; 76.7)	3.64 (3.46, 3.83; 0.50)	586.7 (559.4, 613.9; 68.9)	3.89 (3.70, 4.08; 0.47)
Molecular layer	624.6 (601.6, 647.7; 62.9)	3.69 (3.55, 3.83; 0.38)	581.2 (561.9, 600.4; 48.6)	3.86 (3.71, 4.00; 0.36)
GC‐DG	326.1 (314.4, 337.8; 31.9)	1.93 (1.86, 1.99; 0.18)	300.9 (291.4, 310.4; 24.0)	2.00 (1.93, 2.06; 0.16)
CA1	696.5 (667.2, 725.8; 79.8)	4.21 (3.94, 4.29; 0.47)	641.6 (618.8, 664.3; 57.4)	4.26 (4.09, 4.42; 0.42)
CA2/3	228.4 (217.3, 239.6; 30.4)	1.35 (1.29, 1.41; 0.16)	211.9 (202.2, 221.6; 24.5)	1.40 (1.34, 1.46; 0.15)
CA4	273.6 (263.7, 283.5; 27.1)	1.62 (1.56, 1.67; 0.15)	256.5 (247.8, 265.2; 22.1)	1.70 (1.64, 1.76; 0.15)
Fimbria	91.9 (83.4, 100.4; 23.3)	0.54 (0.49, 0.59; 0.13)	81.6 (76.5, 86.8; 13.0)	0.54 (0.51, 0.58; 0.09)
HATA	66.8 (63.0, 70.5; 10.3)	0.39 (0.37, 0.42; 0.06)	59.2 (56.4, 62.0; 7.1)	0.39 (0.37, 0.41; 0.05)

Note

Values are mean, 95% confidence interval (95% CI) and standard deviation (*SD*) of estimated volume mm^3^ and ratio scale. * indicates *p* < .05. GC‐DG, granule cell layer of dentate gyrus; CA, Cornu Ammonis; HATA, hippocampal‐amygdaloid transition area; Vol, volume (mm^3^); TIV, total intracranial volume. The results of the analysis of covariance using TIV and age as covariates showed a significant gender difference in left GC‐DG (*p* = .01) and left fimbria volumes (*p* = .02), respectively.

### 3.2 Regression analysis for CRR and the whole brain subcortical region volumes

**Figure 2:**
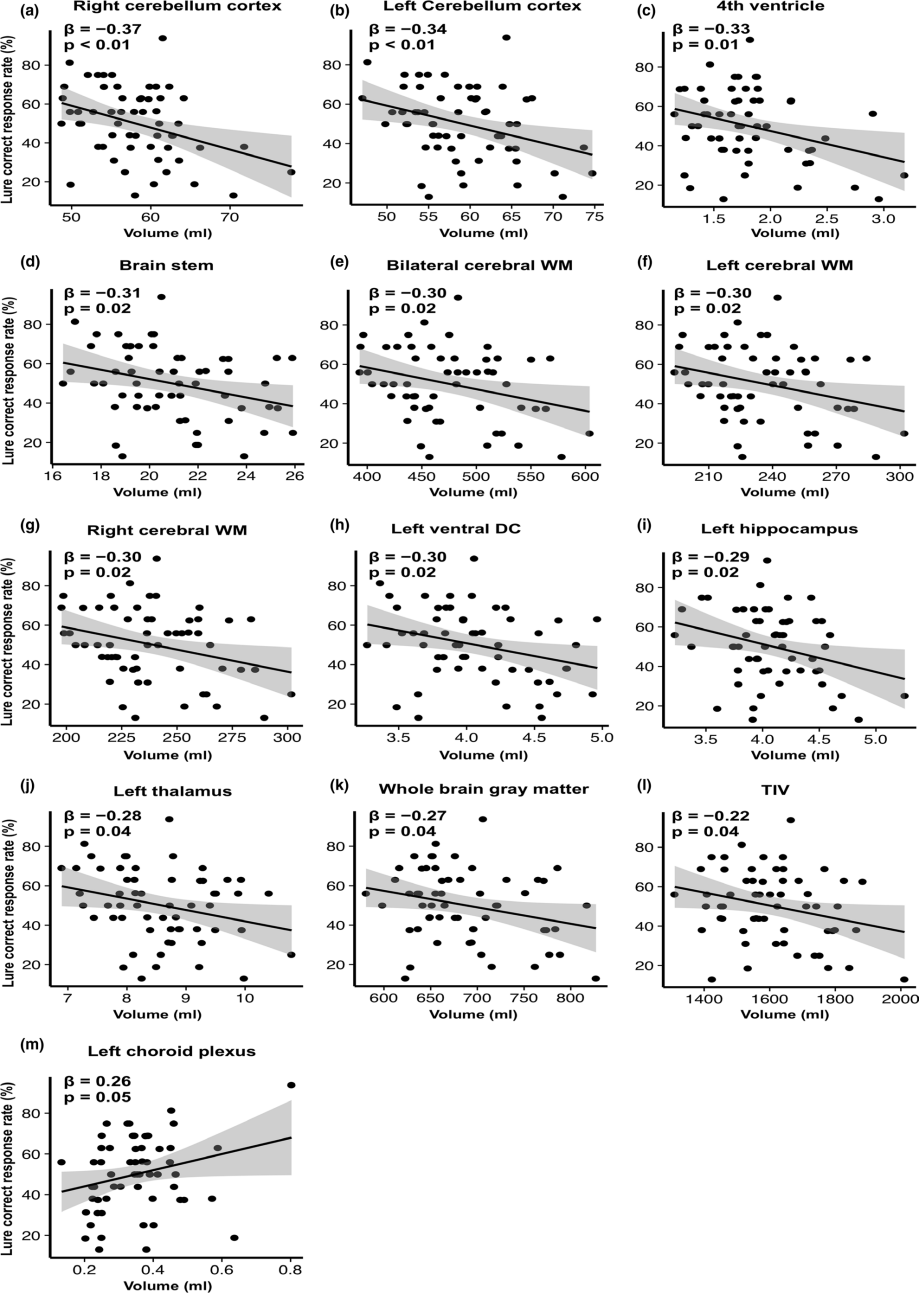
Scatter plot of segmentation brain area showing significant association with the lure task. Note, scatter plot (**a** to **m**) showing significant association by simple linear regression analysis with correct response rate of the lure task and segmented brain area in healthy young subjects (*n* = 58). Each brain area was displayed in descending order of β value. WM, white matter; DC, diencephalon; TIV, total intracranial volume; β indicates standard partial regression coefficient. The solid line represents a linear approximation and 95% confidence interval for regression line shaded gray. Except for the left choroid plexus (**m**), there was a negative association, especially the bilateral cerebellum (**a**, **b**)

**Figure 3:**
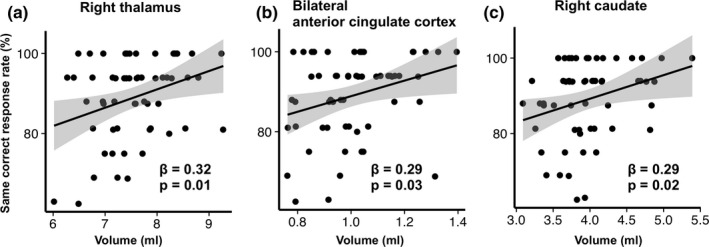
Scatter plot of segmentation brain area showing significant association with the same task. Note, scatter plot (**a** to **c**) showing significant association by simple linear regression analysis with correct response rate of the same task and segmented brain area in healthy young subjects (*n* = 58). Each brain area was displayed in descending order of β value. β indicates standard partial regression coefficient. The solid line represents a linear approximation and 95% confidence interval for regression line shaded gray. The volume of right thalamus, bilateral anterior cingulate cortex, and right caudate nucleus showed positive association with correct response rate to same task (**a** to **c**)

A simple linear regression analysis on the CRR for the memory task and the volumes of the whole brain and subcortical region in all study subjects demonstrated that the lure task performance was negatively correlated with the total intracranial volume, whole brain gray matter volume in bilateral cortical and subcortical regions, volumes in bilateral cerebral white matter, brain stem, fourth ventricle, bilateral cerebellar cortices, left hippocampus, left thalamus, and left ventral diencephalon, while it was positively correlated with the volume in the left choroid plexus (Figure 2). No statistical associations were observed between the CRR for the lure task and other brain regions. The CRR for the same task was positively correlated with bilateral anterior cingulate cortex, right caudate nucleus, and right thalamus (Figure 3, *p* < .05). An analysis using the automatic subcortical segmentation (Aseg) atlas on the whole brain demonstrated that the smaller the volume of the left hippocampus, the higher the CRR in the lure task. There were no significant association about memory task scores and the volume of 46 regions in the multiple comparison correction (Data S3).

### 3.3 Regression analysis for CRR and the all subfield volumes

**Figure 4:**
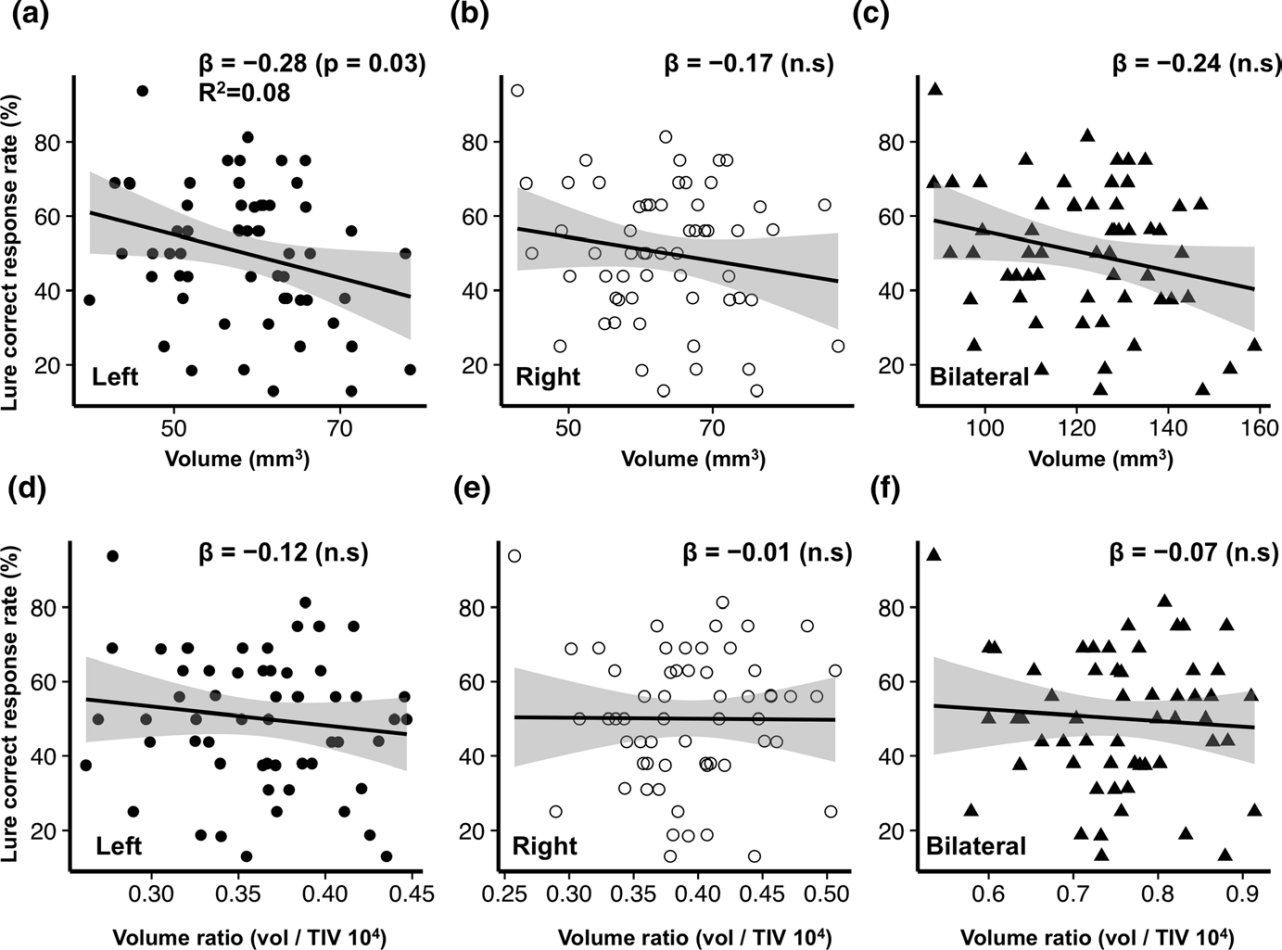
Scatter plot (**a** to **f**) of hippocampal–amygdaloid transition area (HATA) volume and the lure correct response rate (%) with volume ratio analyzed by standard resolution T1 data. Note, the x‐axis indicates the HATA volume mm^3^ (**a** to **c**) and volume ratio (volume/ total intracranial volume; TIV 10^4^) (**d** to **f**). The y‐axis indicates the lure correct response rate (%). The black circle indicates the left HATA, the white circle indicates the right HATA, and the black triangle indicates the bilateral HATA, respectively. β indicates standard partial regression coefficient; R^2^, coefficient of determination; n.s, not significant. The solid line represents a linear approximation and 95% confidence interval for regression line shaded gray. Note, only the left HATA volume showed significant negative association with the lure correct response rate (**a**)

To closely analyze the causal relationships between the hippocampal volume and memory task, simple regression analysis and multiple regression analysis were conducted on the subfield volumes of hippocampus and perihippocampus, the CRR in the lure task, and the CRR in the same task. The simple linear regression analysis revealed that only the volume in the left HATA was negatively correlated with the CRR in the lure task (β = −0.28, *p* = .03, *F* statistic = 4.76, *R* ^2^ = 0.08), but not in other areas with the memory task (Data S4). In the analysis using volume ratio scale, left HATA was no significant relationships (Figure 4). A multiple regression analysis showed no associations between any volumes of the subfields of hippocampus and perihippocmapus, and the CRR in the memory task (Data S5). Analysis using with high‐resolution T2 data simple linear regression analysis also showed a significant negative association only with the HATA in the lure task (Data S6,S7; left volume, β = −0.32, *p* = .01, *F* statistic = 6.33, *R* ^2^ = 0.10; right volume, β = −0.27, *p* = .04, *F* statistic = 4.51, *R* ^2^ = 0.07; bilateral volume, β = −0.32, *p* = .01, *F* statistic = 6.60, *R* ^2^ = 0.11) and multiple regression analysis showed a significant association only with bilateral HATA (β = −0.30, *p* = .05, *F* statistic = 2.93, *R* ^2^ = 0.18) in the lure task (Data S8). In the same task, a simple linear regression analysis showed no significant association (Data S7), and multiple regression analysis showed a significant association with left Hippocampal tail (β = −0.29, *p* = .04, *F* statistic = 2.39, *R* ^2^ = 0.15) and left molecular layer (β = −0.34, *p* = .03, *F* statistic = 2.46, *R* ^2^ = 0.16). There were no significant association about memory task scores and the volume of 12 regions in the multiple comparison correction (Data S4,S5,S7,S8).

## 4 DISCUSSION

### 4.1 Relationship between pattern separation and brain volumes

In this study, a volumetric analysis was undertaken in young healthy individuals aged 40 or younger to investigate the causal association between the hippocampal function and the volumes of the hippocampus and the whole brain, and we noted two things. Firstly, the analysis indicated that there are no direct relationships between the size of the hippocampal subfields and the pattern separation ability. Secondly, the analysis revealed that there were negative causal relations between the pattern separation and the sizes of bilateral cerebral white matter, bilateral cerebellar white matter, left thalamus, left ventral diencephalon, and brain stem. Since volumes in cerebral regions are correlated with the sizes of the body and head, the hippocampal volume must be standardized and results may vary, it has been suggested, depending on this standardization method (Van Petten, 2004). An analysis on the association between the hippocampal volume and memory performance must be carefully performed because TIV as well as gender and age can affect the results. In this analysis, we limited the subjects to right‐handed young people and carried out the analysis by adjusting factors like TIV, as well as gender and age.

### 4.2 The associations between the pattern separation ability and the volumes of the subfields of the hippocampus and perihippocampus

Our results indicated that males had larger volumes in the left DG and the left fimbria than females, which nonetheless did not affect the CRR in the memory task and the response time. No significant causal relations were found between the pattern separation ability and the volumes of the hippocampal subfields. An analysis on the whole brain demonstrated a causal association between the volume of the left hippocampus and the CRR of the pattern separation task; this is believed to be due to the link between the volume in the HATA and the CRR in the task.

In the present study, simple linear regression analysis showed that only the left HATA had a negative correlation with pattern separation. In addition, bilateral HATA was also related to, in multiple regression analysis of covariates TIV, age, and gender with additional high‐resolution T2 data. Anatomically, HATA is composed of extremely densely populated cells adjacent to CA1 and forms a transitional bridge between the head of the hippocampus and caudal amygdala (Fudge et al., 2012). Furthermore, HATA and CA1 display different patterns with acetylcholinesterase staining (Rosene & Van Hoesen, 1987). The interconnection between the amygdala and hippocampal subfields is robust and complicated. It has been established that this interconnection is mediated primarily by glutamatergic pyramidal cells, and more recent studies suggested the involvement of GABAergic projection neurons (Fudge et al., 2012). Further, it has been reported that DG is a target for noradrenergic neurons via the amygdala, as the noradrenergic activity enhances the DG function and is positively correlated with the pattern separation ability (Segal et al., 2012). Neural activity in DG can be adjusted through electric stimulation on the basolateral amygdala, and though there is anatomically no direct pathway between DG and the amygdala, some studies demonstrated that there is an indirect pathway (Nakao et al., 2004). It has been reported that stress inhibits neurogenesis (Malberg & Duman, 2003; Wu et al., 2014), and conversely, the increased prevalence of neurogenesis reduced anxiety and depression‐like behavior in mice, while it did not affect hypothalamic–pituitary–adrenal axis regulation and it instead affected the psychology of mice through an independent channel (Hill et al., 2015). HATA is a relay region that serves as a bridge between the hippocampus and amygdala. It may adjust neurogenesis with pattern separation and mental function by forming an indirect pathway with the hippocampal DG. By reconsidering the association between the volumetric MRI in the HATA and stress or pattern separation, the HATA volume might serve as an important index for the prevention of mental illnesses.

### 4.3 The associations between pattern separation performance and the whole brain cortical and subcortical volume

This study demonstrated that the smaller the volumes in the bilateral white matter regions, bilateral cerebellar cortices, left thalamus, left ventral diencephalon, and brain stem, the greater the pattern separation ability. Among these regions, a particularly strong association was noted in bilateral cerebellar cortices. In addition, while a significant association was observed between the whole brain gray matter volume including the cerebellum and basal ganglia and the CRR in the lure task, no such correlations were noted in the bilateral cerebral gray matter regions, bilateral subcortical gray matter regions. This implies strong causal relevance of the bilateral cerebellar cortices. The associations between the cerebellar cortex volume and the pattern separation ability suggest that the structure of the cerebellar cortex affects pattern separation. The cerebellum is closely associated with the pattern separation function (Shiroma et al., 2015). Patients who experienced mechanical compression on their posterior cerebellum Crus I due to cerebellopontine angle tumors including vestibular schwannoma exhibited a decline in the pattern separation ability, while the ability improved when tumorectomy released the compression (Shiroma et al., 2015). Even in healthy subjects, CRR in the lure task for the pattern separation ability and the volume of cerebellum Crus I may have a specifically stronger relationship than other areas of the cerebellar cortex, but it requires further studies.

### 4.4 Limitations

There are a few limitations in interpreting this study results. Firstly, this study is a cross‐sectional analysis of 58 subjects aged 18 to 40 (31 males and 27 females), and caution must be taken in interpreting the gender difference because of the small sample size particularly for gender comparison. A longitudinal analysis using big data is required. Secondly, while the pattern separation task obtained as the behavioral index showed a standard distribution, the results of the pattern completion were distributed in a high score range and did not show a standard distribution, this might have prevented us from elucidating associations with volumes.

## CONFLICT OF INTEREST

The authors declare no potential conflict of interest.

## AUTHOR CONTRIBUTIONS

SI: Designed and administered this study and raised integrated hypothesis and wrote the manuscript. MN: Acquisition of MRI and behavioral data. RU: Neuroimaging analysis and wrote the manuscript. All authors edited the manuscript.

### Peer Review

The peer review history for this article is available at https://publons.com/publon/10.1002/brb3.1878.

## Supporting information

Supplementary Material

## Data Availability

Requests for the data can be sent to ishogo@med.u‐ryukyu.ac.jp.
