# Supplementary material for: Analysis of human hippocampal volumetry in relation to pattern separation ability in healthy young subjects
Source: Brain Behav. 2020 Oct 23;10(12):e01878. doi: 10.1002/brb3.1878 (PMC7749596; doi:10.1002/brb3.1878)
Supplement: Supplementary file 1 — Supplementary Material [file BRB3-10-e01878-s001.docx]

Supplementary 1.


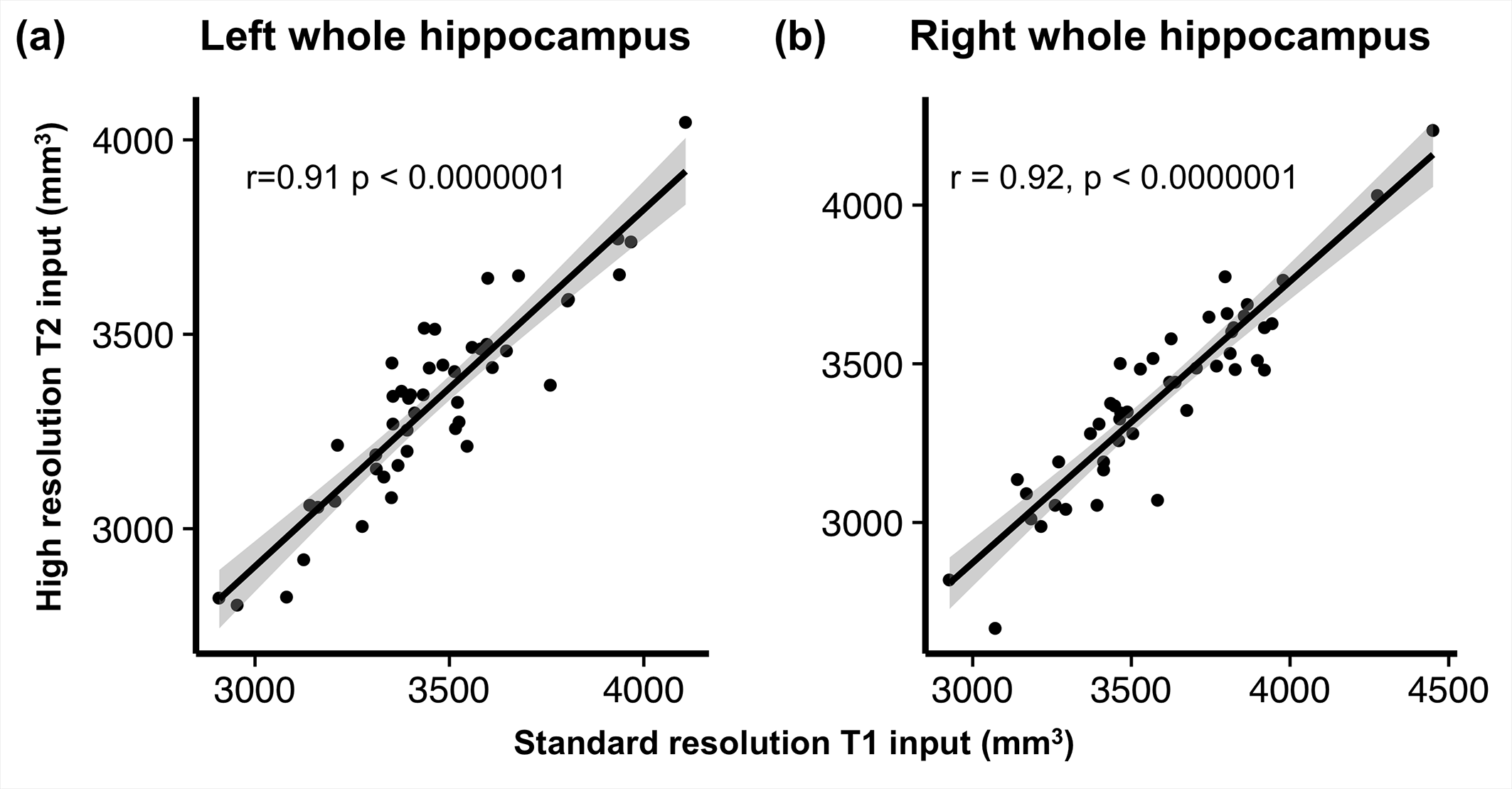

Note, scatter plots indicates whole hippocampus volume estimated on high-resolution T2 input vs standard-resolution T1 input in 47 young healthy subjects (mean age 25.0 ± 4.7 years, 22 males, 25 females). The solid line represents a linear approximation and 95% confidence interval for regression line shaded gray. Whole hippocampus and subfield volumes estimated by Freesurfer 6 with high-resolution T2 and standard-resolution T1, showed significant correlation in all area. r, pearson’s correlation coefficient; 95% CI, 95% confidence interval; P, P value. GC-DG, granule cell layer of dentate gyrus; CA, Cornu Ammonis; HATA, hippocampal-amygdaloid transition area.

Supplementary 2.

Characteristic of the subfield volumes of hippocampus and perihippocampus segmented using high-resolution T2

Note, values are mean, 95% confidence interval (95% CI) and standard deviation (SD) of estimated volume mm^3^ and ratio scale. Of all 58 subjects 47 include high-resolution T2 and 11 include T1 input. * indicates p < 0.05. GC-DG, granule cell layer of dentate gyrus; CA, Cornu Ammonis; HATA, hippocampal-amygdaloid transition area; Vol, volume (mm^3^); TIV, total intracranial volume. The results of the analysis of covariance using TIV and age as covariates showed a significant gender difference in left GC-DG (P=0.05) and left fimbria volumes (P=0.02), respectively.

Supplementary 3. Simple linear regression analysis for memory task score and Automated segmentation volumes (Aseg)

Note, β indicates standard partial regression coefficient; 95% CI, confidence interval; t, t-value; P, P-value; P**^a^**, The values were corrected by Benjamini-Krieger-Yekutieli’s method for multiple comparisons; *, p< 0.05; **, p< 0.01. TIV, total intracranial volume; GM, gray matter; WM, white matter; CSF, cerebrospinal fluid; DC, Diencephalon. The right and left cerebellum cortex showed a strong negative association with the lure task (p < 0.01).

Supplementary 4. Simple linear regression analysis for memory task score and the subfield volumes of hippocampus and perihippocampus segmented using standard-resolution T1

Note, simple linear regression analysis was performed between memory task score （correct response rate of lure task and same task） and whole hippocampus or each subfield volumes. β indicates standard partial regression coefficient; 95% CI, confidence interval; t, t-value; P, P-value; P**^a^**, The values were corrected by Benjamini-Krieger-Yekutieli’s method for multiple comparisons; *, p< 0.05; **, p< 0.01. GC-DG, granule cell layer of dentate gyrus; CA, Cornu Ammonis; HATA, hippocampal-amygdaloid transition area. Only the left HATA indicated significant negative association with the lure correct response rate (P = 0.03).

Supplementary 5. Multiple regression analysis for memory task score and the subfield volumes of hippocampus and perihippocampus, TIV, age and gender segmented using standard-resolution T1

(continued)

(continued)

Note, the model in multiple regression analysis was analyzed memory task score (correct response rate of lure task and same task) as the dependent variable and whole hippocampus or subfield volume, TIV, age and gender as the explanatory variable. β indicates standard partial regression coefficient; 95% CI, confidence interval; t, t-value; P, P-value; P**^a^**, The values were corrected by Benjamini-Krieger-Yekutieli’s method for multiple comparisons; *, p< 0.05; **, p< 0.01. GC-DG, granule cell layer of dentate gyrus; CA, Cornu Ammonis; HATA, hippocampal-amygdaloid transition area. There was no significant causal association with memory task in all areas.

Supplementary 6.


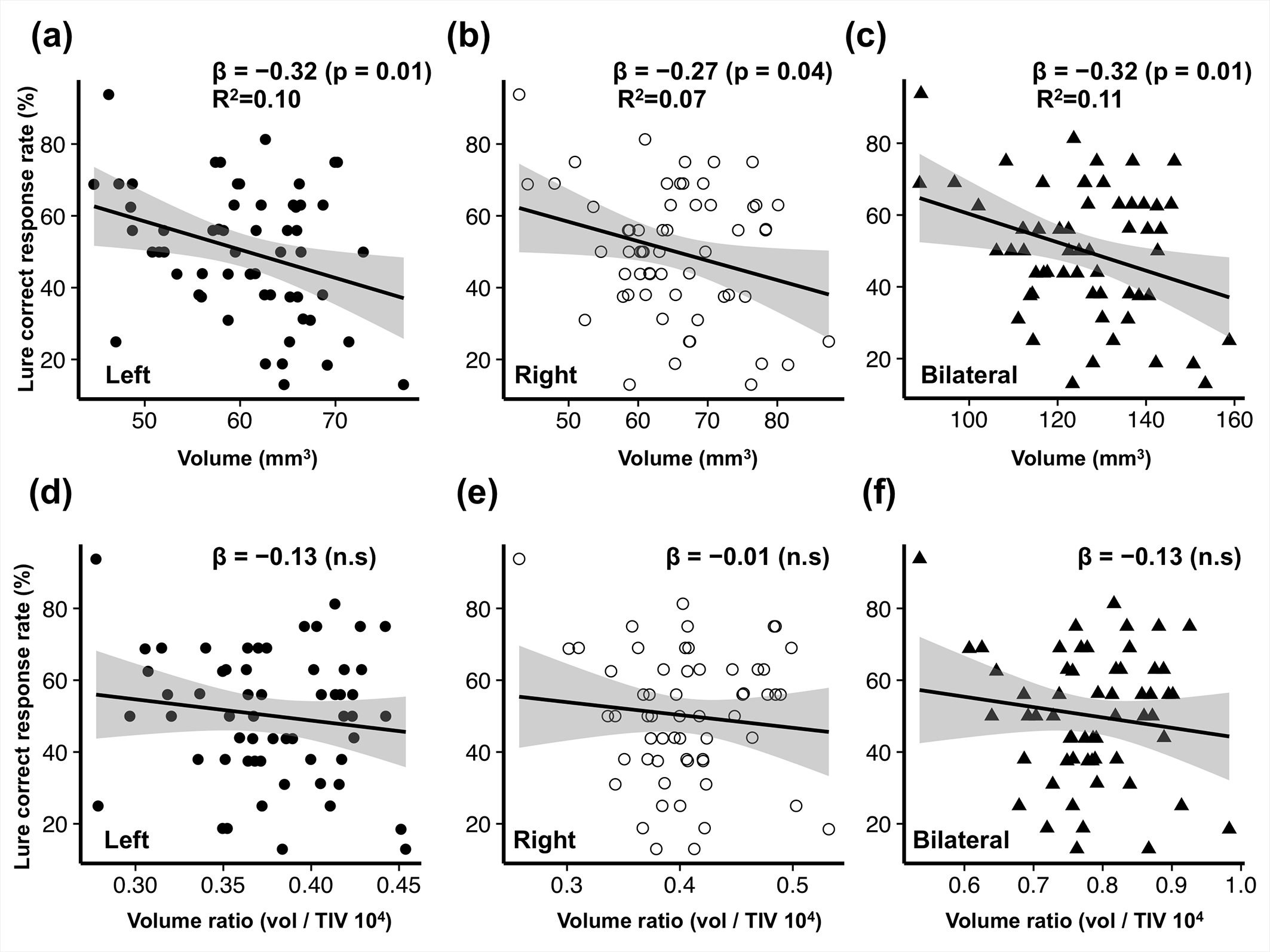


Scatter plot (**a** to **f**) of hippocampal-amygdaloid transition area (HATA) volume and the lure correct response rate (%) with volume ratio analyzed by high-resolution T2 data (N = 58, Of all 58 subjects 47 include high-resolution T2 and 11 include T1 input). Note, the X-axis indicates the HATA volume mm^3^ (**a** to **c**) and volume ratio (volume / Total Intracranial Volume; TIV 10^4^) (**d** to **f**). The y-axis indicates the lure correct response rate (%). The black circle indicates the left HATA, the white circle indicates the right HATA, and the black triangle indicates the bilateral HATA, respectively. β indicates standard partial regression coefficient; R^2^, coefficient of determination; n.s, not significant. The solid line represents a linear approximation and 95% confidence interval for regression line shaded gray. Note, only the HATA volume showed significant negative association with the lure correct response rate (**a** to **c**).

Supplementary 7. Simple linear regression analysis for memory task score and the subfield volumes of hippocampus and perihippocampus segmented using high-resolution T2

Note, simple linear regression analysis was performed between memory task score (correct response rate of lure task and same task) and whole hippocampus or each subfield volumes. Of all 58 subjects 47 include high-resolution T2 and 11 include T1 input. β indicates standard partial regression coefficient; 95% CI, confidence interval; t, t-value; P, P-value; P**^a^**, The values were corrected by Benjamini-Krieger-Yekutieli’s method for multiple comparisons; *, p< 0.05; **, p< 0.01. GC-DG, granule cell layer of dentate gyrus; CA, Cornu Ammonis; HATA, hippocampal-amygdaloid transition area. Only the HATA indicated significant negative association with the lure correct response rate (P < 0.05).

Supplementary 8. Multiple regression analysis for memory task score and the subfield volumes of hippocampus and perihippocampus, TIV, age and gender segmented using high-resolution T2

(continued)

(continued)

Note, the model in multiple regression analysis was analyzed memory task score (correct response rate of lure task and same task) as the dependent variable and whole hippocampus or subfield volume, TIV, age and gender as the explanatory variable. Of all 58 subjects 47 include high-resolution T2 and 11 include T1 input. β indicates standard partial regression coefficient; 95% CI, confidence interval; t, t-value; P, P-value; P**^a^**, The values were corrected by Benjamini-Krieger-Yekutieli’s method for multiple comparisons; *, p< 0.05; **, p< 0.01. GC-DG, granule cell layer of dentate gyrus; CA, Cornu Ammonis; HATA, hippocampal-amygdaloid transition area. There was significant relationship between bilateral HATA (P = 0.05) and the lure task, and between left hippocampal tail (P = 0.04), molecular layer (P = 0.03) and the same task, respectively.
